# Global prevalence of post-COVID-19 condition: a systematic review and meta-analysis of prospective evidence

**DOI:** 10.24095/hpcdp.45.3.02

**Published:** 2025-03

**Authors:** Mohamed Kadry Taher, Talia Salzman, Allyson Banal, Kate Morissette, Francesca R. Domingo, Angela M. Cheung, Curtis L. Cooper, Laura Boland, Alexandra M. Zuckermann, Muhammad A. Mullah, Claudie Laprise, Roberto Colonna, Ayan Hashi, Prinon Rahman, Erin Collins, Tricia Corrin, Lisa A. Waddell, Jason E. Pagaduan, Rukshanda Ahmad, Alejandra P. Jaramillo Garcia

**Affiliations:** Evidence Synthesis and Knowledge Translation Unit, Centre for Surveillance and Applied Research, Health Promotion and Chronic Disease Prevention Branch, Public Health Agency of Canada, Ottawa, Ontario, Canada; 2 School of Epidemiology and Public Health, University of Ottawa, Ottawa, Ontario, Canada; 3 Department of Medicine and Joint Department of Medical Imaging, University Health Network and Sinai Health System, University of Toronto, Toronto, Ontario, Canada; 4 Toronto General Hospital Research Institute and Schroeder Arthritis Institute, Toronto, Ontario, Canada; 5 Department of Medicine, University of Ottawa; Ottawa Hospital Research Institute, Ottawa, Ontario, Canada; 6 Infectious Disease and Vaccination Programs Branch, Centre for Communicable Diseases and Infection Control, Public Health Agency of Canada, Ottawa, Ontario, Canada; 7 Department of Social and Preventive Medicine, School of Public Health, Universit de Montral, Montral, Quebec, Canada; 8 Population Health Modelling Unit, Centre for Surveillance and Applied Research, Health Promotion and Chronic Disease Prevention Branch, Public Health Agency of Canada, Ottawa, Ontario, Canada; 9 Public Health Risk Sciences Division, National Microbiology Laboratory, Public Health Agency of Canada, Guelph, Ontario, Canada; 10 Risk Assessment Division, Centre for Surveillance, Integrated Insights and Risk Assessment, Data, Surveillance and Foresight Branch, Public Health Agency of Canada, Ottawa, Ontario, Canada

**Keywords:** post-COVID-19 condition, post-COVID condition, PCC, post-acute sequelae of COVID-19, PASC, Long COVID, COVID-19 Long-Hauler, COVID-19 recovery, long-term effects of COVID-19, prevalence, systematic review, prospective studies

## Abstract

**Introduction:**

We investigated the prevalence of new or persistent manifestations experienced by COVID-19 survivors at 3 or more months after their initial infection, collectively known as post-COVID-19 condition (PCC).

**Methods:**

We searched four electronic databases and major grey literature resources for prospective studies, systematic reviews, authoritative reports and population surveys. A random-effects meta-analysis pooled the prevalence data of 22 symptoms and outcomes. The GRADE approach was used to assess the certainty of evidence. PROSPERO CRD42021231476.

**Results:**

Of 20 731 identified references, 194 met our inclusion criteria. These studies followed 483531 individuals with confirmed COVID-19 diagnosis over periods of up to 2 years. Most focused on adults, nearly two-thirds were conducted in Europe and 63% were of high or moderate quality. The supplementary search identified 17 systematic reviews, five authoritative reports and four population surveys that reported on PCC prevalence. Our analysis revealed that more than half of COVID-19 survivors experienced one or more symptoms more than a year after their initial infection. The most common symptoms were fatigue; dyspnea; memory, sleep or concentration disturbances; depression; and pain. Limitation in returning to work was the most common outcome. Prevalence tended to be higher among females, individuals hospitalized during their initial infection and those who experienced severe COVID-19 illness.

**Conclusion:**

PCC presents a significant health burden, affecting some groups more than others. This information will help inform health care system policies and services for people living with PCC and those caring for them.

**Highlights**

- We searched for prospective studies of the prevalence of post-COVID-19 condition (PCC) published up to 15 July 2022 and systematic reviews, authoritative reports and population surveys published up to 8 December 2023.
- Through group and subgroup analyses, we pooled prevalence data from 483 531 adults and children with new or persistent symptoms at least 3 months after their confirmed SARS-CoV-2 infection.
- More than 50% of COVID-19 survivors experienced at least one PCC symptom up to 2 years after their initial infection.
- The most common symptoms were dyspnea, fatigue, pain and depression, and the most common outcome was not returning to work.

## Introduction

Almost 5 years after the first reported case of “pneumonia of unknown etiology,”^1^ more than 772 million individuals have been reported as infected with SARS-CoV-2, and COVID-19 disease has contributed to more than 7 million deaths.^2^ While many COVID-19 survivors recover fully from their acute infection, others developed or continue to experience a number of symptoms or outcomes for various periods of time.

The World Health Organization defines post-COVID-19 condition (PCC) as new or persistent symptoms that first occur 3 or more months after confirmed or suspected COVID-19, last for a minimum of 2 months and cannot be attributed to any other cause.^3^,^4^ The most commonly reported symptoms include fatigue, dyspnea, cognitive dysfunction, memory or sleep disturbances, cough, tachycardia, pain, disturbed smell or taste, depression, anxiety and fever.^3^-^6^

Although health authorities were mostly able to record numbers of COVID-19 cases, estimating the number of individuals who experience PCC symptoms is difficult, largely because of the lack of a universally accepted definition of PCC, which has more than 200 primary symptoms or conditions, also with different definitions and assessment methods.^3^-^6^ Consequently, reported PCC prevalence varies widely, from less than 1% to more than 50%, across studies.^7^-^12^ Also contributing to this variation is the use of estimates that include both suspected and confirmed cases, that are based on different study designs and that apply different outcome assessment methods.

Statistical models projected that, by the end of 2021, approximately 145million individuals, representing 3.7% of the nearly 4 billion people estimated to have been infected with COVID-19, could have experienced PCC.^13^ These models also projected that 15.1% of this population might continue to experience these symptoms for more than 1 year after their initial infection.^13^,^14^

Results from recent population surveys conducted to assess the overall prevalence of PCC symptoms among adults vary from 14.3% in the USA^15^ to 6.8% in Canada^16^ and 4.7% in Australia.^17^ A 2023 national survey found that 1.6 million individuals in the United Kingdom, or 2.6% of the total population, reported experiencing PCC symptoms.^11^ Multiple studies have found that females were more likely than males to report PCC symptoms.^11^,^15^,^17^,^18^

A 2023 Canadian study projected the burden of PCC on the health care system to be between CAD 7.8 and 50.6 billion, with the cost per case between CAD 1675 and 7340 and the reduction in quality-adjusted life years between 0.047 and 0.206 during the first year after the initial infection.^19^ A 2022 US study estimated annual PCC health care costs to be between USD 43 and 172billion and lost income due to PCC to be between USD 101 and 430 billion, to a total loss of USD 140 to 600 billion annually.^7^ These estimates exclude costs related to disability services, social services and caregiver income loss.^7^

The objective of this current review is to systematically identify, examine and analyze the prospective epidemiological evidence on the prevalence of predefined PCC symptoms and outcomes that emerged or continued to persist 3 or more months after confirmed COVID-19 diagnosis.

## Methods

***Systematic review registration***

The review protocol was registered in PROSPERO, the international prospective register of systematic reviews (CRD42021231476), and followed Cochrane guidance^20^ and Preferred Reporting Items for Systematic Reviews and Meta-Analyses (PRISMA) guidance.^21^

***Inclusion criteria***

This systematic review focuses on prospective studies (cohort studies and clinical trials) because they establish exposure to COVID-19 prior to development of PCC, which increases our certainty on the causative relationship between the two; because of their robust methodology; and because of their minimal susceptibility to recall bias.^22^-^24^ Included were primary, prospective, peer-reviewed studies, published in English or French, with a minimum of 50 participants who reported new or persistent symptoms or outcomes 12 or more weeks after the onset of a confirmed COVID-19 diagnosis. Excluded were studies that recruited participants based on existing PCC symptoms or outcomes, or after the acute phase resolution (4 weeks).

Symptoms or outcomes were selected via consensus reached during consultations with patient representatives, clinical experts and policy makers. These symptoms and outcomes were based on patient concerns, clinical relevance and impact on health care service delivery. The symptoms included fatigue, dyspnea, pain, cognitive impairment, major cardiovascular events, psychopathologies and sleep disturbances, while outcomes included mobility issues and functional impairment. (Refer to Appendix A in Supplementary Material I for the study selection and data collection process, and a list of excluded studies.)

***Search strategy***

We implemented a comprehensive search strategy, adapted from the National Institute for Health and Care Excellence (NICE) guideline on long COVID,^25^ to identify original prospective studies investigating the prevalence of PCC symptoms and outcomes in people, irrespective of their sex, age, race or ethnicity, country of residence or any other factor. A librarian conducted a peer review and determined that this search strategy aligned with our search criteria. 

The initial search, undertaken on 15 July 2022, retrieved studies published between 22 October 2020 and 15 July 2022 from MEDLINE, Embase, Cochrane CENTRAL, PsycINFO and major grey literature resources. We conducted an extended search, using the same search strategy, on 13 to 31 March 2023, to identify systematic reviews, authoritative reports and population surveys that reported evidence published after 15 July 2022 to ensure a comprehensive and up-to-date contextual understanding. We continued monitoring for authoritative reports and population surveys through 8 December 2023. 

***Study selection***

Using the DistillerSR application (DistillerSR Inc., Ottawa, ON, CA),^26^ we developed and piloted a title and abstract screening form and a full-text examination form. These forms were piloted by multiple reviewers (AH, AMZ, CL, EC, FRD, KM, LB, AB, MKT, RC, TC and TS) and subsequently adjusted before their full-scale implementation. In each phase, two reviewers independently applied these forms to each study to assess conformance with the inclusion criteria. A single reviewer was sufficient to screen study titles and abstracts for potential relevance and move a reference to full-text screening, whereas two reviewers were required to exclude a study. Two reviewers were also required to exclude a citation or promote it to the next level during full-text examination. Any disagreements at the full-text screening stage were resolved through discussion.

***Data extraction***

Data extraction forms were created in advance of the review using DistillerSR.^26^ These forms were used to capture key study characteristics, patient demographics and outcome data (outlined in Table 1, Appendix B in Supplementary Material I and in Supplementary Material II). One reviewer (AH, AMZ, CL, EC, FRD, KM, LB, AB, MKT, RC, TC or TS) conducted the initial data extraction for each study, while a second cross-checked the extracted information for accuracy and completeness.

**Table 1:** Key study characteristics, patient demographics and outcome data in included studies (n = 194)

Major characteristics	Study design: cohort / randomized controlled trial
	Case management: hospital-based (ICU/ward) / community-based (outpatient/ambulatory) / mixed
	Diagnosis: lab / clinical / lab + clinical
Patient demographics	Country of residence
	Population group: adults / children / all ages; males / females
Symptoms^a^	Dyspnea
	Fatigue
	Palpitations/tachycardia
	Pain: arthralgia; chest pain; headache; myalgia
	Cognitive impairment: brain fog; cognitive impairment (unspecified); concentration disturbance; memory disturbance
	Clinical psychopathologies: anxiety; depression; PTSD
	Health-related quality of life: sleep disturbance (unspecified); insomnia
Outcomes^a^	Mobility problems; limitations in returning to work^b^; difficulties with self-care; difficulties performing daily activities

**Abbreviations:** PTSD, posttraumatic stress disorder; ICU, intensive care unit.

^a^ Symptoms and outcomes were selected during consultations with patient representatives, clinical experts and policy makers, and were based on patient concerns, clinical relevance and impact on health care service delivery. 

^b^ “Did not return to work” and “unable to return to work” were reported separately by some studies. Collectively these are described as “limitations in returning to work.”

***Assessment of risk of bias***

We conducted an assessment of risk of bias (ROB) using a modified version of the Joanna Briggs Institute (JBI) appraisal tool for prevalence studies.^27^,^28^ In consultation with the authors of the JBI critical appraisal tool, we omitted some questions to prevent overlap with the criteria for assessing imprecision and indirectness as part of the assessment of certainty of evidence (COE). Each study was assessed for ROB using the following JBI critical appraisal checklist questions: “Was the sample frame appropriate to address the target population?”; “Were study participants sampled in an appropriate way?”; and “Was the response rate adequate, and if not, was the low response rate managed appropriately?”^28^ Each outcome was assessed separately for ROB using the following JBI critical appraisal checklist questions: “Were valid methods used for the identification of the condition?”; and “Was the condition measured in a standard, reliable way for all participants?”^28^ Responses to all the questions were either “yes” or “no.”

The questions were then grouped into three domains: participants (population, sampling and response rate); outcome measures (identification and measurement); and statistics (reported data). Studies fully meeting the criteria within these domains were rated as having a low ROB, those partly meeting the criteria were rated as having a moderate ROB and those not meeting the criteria were rated as having a high ROB. Each study was assessed for ROB by one reviewer and validated by another (AH, AMZ, EC, FRD, KM, LB, AB, PR, RC, TC and TS), with a third resolving any disagreements (MKT). (For more details, refer to Appendix C in Supplementary Material I.)

***Data analysis***

Extracted data were collated, cleaned and standardized using Excel 2019 (Microsoft, Redmond, WA, US).^29^ We introduced a new variable, “onset to follow-up,” to try to harmonize the different follow-up periods across included studies. These follow-up periods describe the time between confirmation of COVID-19 diagnosis, onset of symptoms or patient recovery and follow-up assessment. For those studies that described follow-up assessments as starting from the time of patient recovery, we added 1 week to the reported period. For those studies that described follow-up assessments as starting from the time of hospital discharge, we added 2 weeks to the reported period.

To better understand changes in PCC prevalence over time, we grouped our prevalence data into these four follow-up periods: 12 to 26 weeks, 27 to 39 weeks, 40 to 52 weeks, and more than 1 year. Where a study reported multiple times on an outcome/symptom within the same period, we used the data from the longest follow-up period.

We conducted a series of meta-analyses for prevalence data using a random-effects model to allow for expected heterogeneity of the included studies.^20^,^21^ In our primary analysis, we pooled the data for each symptom and outcome and follow-up period separately across studies. To explore the reasons for heterogeneity, we conducted subgroup analyses by ROB level (low or moderate versus high), study population (hospital-based, community-based, mixed) and continent.

Whenever sufficient data were available, we stratified the prevalence data by sex, severity of disease, case management (hospitalized versus ambulatory) and level of hospital care (intensive care unit [ICU] versus ward) during the initial infection. While we explored stratification by age, race or ethnicity, or pre-existing conditions, analysis was not always feasible because of limited availability of data. If multiple studies examined the same population, we included only the most recent publication in the meta-analysis.

We performed the analyses using the statistical software RStudio version 1.4.1106 (R Foundation for Statistical Computing, Vienna, AT).^30^ We used the “metaprop” function from the “meta” package for the meta-analysis and the “forest.meta” function from the same package to generate the forest plots.

***Assessment of certainty of evidence***

Upon completing the meta-analysis, we assessed the COE for each symptom and outcome using a modified version of the Grading of Recommendations, Assessment, Development, and Evaluations (GRADE) approach^31^,^32^ for prognostic studies (previously adopted by Righy et al.^33^). An experienced assessor (AMZ, FRD, KM, LB, MKT or PR) evaluated the evidence for each symptom or outcome for the total sample across all follow-up periods. These assessments were then validated by another team member; in the event of any discrepancies, discussion among assessors continued until agreement was reached. 

Each symptom and outcome was evaluated across several domains: ROB, inconsistency, indirectness and imprecision. ROB, in particular, was assessed across all studies reporting on a specific symptom or outcome using the GRADE approach. Unlike the JBI ROB tool, which evaluates bias within individual studies and their symptoms and outcomes, the GRADE approach examines ROB across all studies for each symptom and outcome, categorizing it as “not serious,” “serious” or “very serious.”

In addition to ROB, inconsistency was assessed by examining variability in prevalence estimates across studies. Indirectness was evaluated based on the relevance of the study population to the research question, while imprecision was assessed by analyzing sample sizes. Each of these domains contributed to determining the overall COE for each symptom and outcome across the four follow-up periods, classified as “high,” “moderate,” “low” or “very low.” (For more details on the GRADE assessment, refer to Appendix D in Supplementary Material I.)

## Results

**Figure 1:**
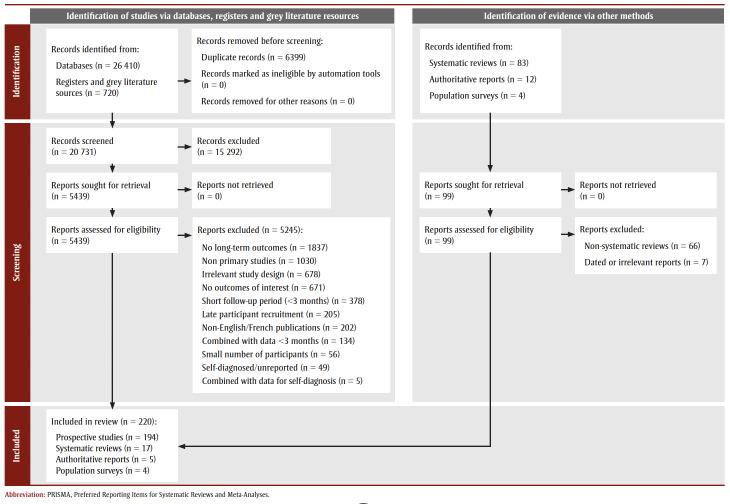
PRISMA 2020 flow diagram of included studies

Our primary search identified 20 731 unique citations from the four databases and grey literature resources. Of these, 194 met the inclusion criteria (see Figure 1). 

The extended search identified 17 systematic reviews, five authoritative reports and four population surveys published between 2021 and 2023.

***Overview of studies***

Except for two clinical trials,^34^,^35^ all of the 194 included studies were observational prospective cohort studies. More than 60% (n = 120) of the included studies were conducted in seven countries, namely Italy,^36^-^65^ China, 34,66-90 Spain,^91^-^112^ the USA,^113^-^127^ France,^128^-^136^ Switzerland^137^-^145^ and the United Kingdom,^146^-^154^ in order of number of retrieved studies. Four percent of the studies (n = 7) were conducted in the Netherlands,^35^,^155^-^160^, 3% (n = 6) in Mexico,^161^-^166^ 2.6% in Brazil,^167^-^171^ Denmark,^172^-^176^ Germany,^176^-^181^ Sweden^182^-^186^ and Turkey^187^-^191^ (n = 5 each) and 2.1% in Belgium^192^-^195^ and India^196^-^199^ (n = 4 each). As well, 1.5% of the studies were conducted in Iran^200^-^202^ and Poland^203^-^205^ (n = 3 each) and 1.0% in Australia,^206^,^207^ Israel, 208,209 Norway,^210^,^211^ Pakistan,^212^,^213^ Russia^214^,^215^ and South Korea^216^,^217^ (n = 2 each). A single study was conducted in Austria,^218^ Chile,^219^ Iraq,^220^ Ireland,^221^ Japan,^222^ Saudi Arabia,^223^ Serbia^224^ and Singapore^225^ each. Two studies were conducted in multiple countries.^226^,^227^ None of the included studies were conducted in Canada.

***Follow-up periods***

Most studies (n=106) covered the 12- to 26-week period following the initial infection. Fewer studies covered periods of 27 to 39 weeks (n=39), 40 to 52 weeks (n=22) or more than 1 year (n=25).

***Patient demographics***

The retrieved studies examined a total of 483 531 individuals with confirmed COVID-19 over follow-up periods of up to 2 years. Infection of 82% of participants in the included studies was confirmed by positive polymerase chain reaction (PCR) test; confirmation of infection of the remaining 18% relied on clinical diagnosis or a combination of clinical and laboratory diagnoses. 

As many as 95% of studies (n = 184) included only adult participants (> 18 years). Seven studies reported data for both adults and children combined,^78^,^84^,^128^,^151^,^175^,^183^,^196^ two studies focused exclusively on children and adolescents (≤18 years)^185^,^214^ and one study provided separate data for children.^215^ More than two-thirds (70%; n = 136) of studies included participants who were hospitalized during their initial infection; 8% included nonhospitalized (or ambulatory) patients; and 22% included both populations. We estimated the fair representation of females to range between 45% and 55% of the total study population. In 51% of the included studies, females made up less than 45% of the study sample. (More details on the excluded and included studies are provided in appendices A and B in Supplementary Material I, respectively.)

***Risk of bias***

Assessment of the included studies determined that 57% had moderate ROB, 39% had high ROB and 5% had low ROB. For most symptoms and outcomes, more than half of the reporting studies were considered to be of low or moderate ROB for these specific symptoms and outcomes. However, for anxiety, depression, posttraumatic stress disorder (PTSD), memory disturbance and mobility problems, more than half of the studies had high ROB. (ROB assessments are detailed in appendices B and C in Supplementary Material I.)

***Certainty of evidence***

The COE of 57% of assessments of symptoms or outcomes for the total sample across all follow-up periods was very low, of 39% was low and of 3% was moderate. Regarding ROB, most analyses demonstrated serious ROB, with 49% reflecting a full-point reduction and 14% reflecting a half-point reduction in certainty. In addition, 32% of the analyses were categorized as having a very serious ROB. In terms of inconsistency, the majority of assessments were also rated as serious, with 53% reflecting a full-point reduction and 31% reflecting a half-point reduction. Indirectness was also a significant factor, as 91% of the assessments were rated as serious due to the predominance of hospital-based populations in the supporting studies. Only 8% of analyses were supported by studies examining more balanced populations, such as community-based or mixed settings. Imprecision, assessed using the optimal information size criterion, was found to be non-serious in 93% of the analyses. However, 6% of the analyses were rated as serious due to unmet information size criterion thresholds. (For more information regarding the GRADE assessment, refer to Figures 3–6 and Appendix D in Supplementary Material I.)

***Symptoms and outcomes***

Across nearly all follow-up periods, more than half of the COVID-19 survivors reported experiencing one or more PCC symptoms: 56.5% (n = 14 615) at 12 to 26 weeks; 50.9% (n = 2764) at 27 to 39 weeks; and 77.6% (n = 2337) at over 1 year. At 40–52 weeks, the percentage reporting experiencing one or more PCC symptoms was lower, at 32.6% (n = 1198). 

Fatigue (n = 101) and dyspnea (n = 98) were commonly reported in studies covering all follow-up periods. Other symptoms and outcomes were headache (n = 65), myalgia (n = 53), chest pain (n = 48) and palpitations/tachycardia (n = 41), although their relative rankings varied over time. Limitations in returning to work (n = 12) were the most commonly reported outcomes related to functional impairment in studies. (For an overview of the most prominent prevalence estimates, the number of studies contributing to each symptom or outcome as well as the level of heterogeneity of the studies, see Table2; for more details, refer to Supplementary Material II.)

**Table 2:** Pooled prevalence estimatesa for the assessed PCC symptoms and outcomesb with GRADE-assessed certainty of evidence at different follow-up periods

Symptom / outcome	Value	Follow-up period			
		12–26 weeks	27–39 weeks	40–52 weeks	> 1 year
Symptom					
General					
≥1 symptoms	Pooled prevalence estimate, % (95% CI)	56.52 (47.18–65.42)	50.89 (33.53–68.03)	32.64 (19.64–49.00)	77.64 (52.23–91.69)
	I^2^, %	99	98	96	99
	No. of studies, n	32	9	4	6
	COE	Very low	Very low	Low	Very low
Dyspnea	Pooled prevalence estimate, % (95% CI)	20.55 (13.64–29.76)	14.81 (11.41–19.01)	16.06 (11.60–21.82)	15.62 (8.76–26.31)
	I^2^, %	100	94	96	99
	No. of studies, n	61	22	15	18
	COE	Low	Low	Low	Very low
Fatigue	Pooled prevalence estimate, % (95% CI)	29.90 (19.20–43.50)	28.38 (21.12–36.96)	30.70 (19.40–44.90)	26.90 (18.20–37.70)
	I^2^, %	100	98	98	99
	No. of studies, n	62	26	12	19
	COE	Very low	Very low	Low	Very low
Palpitations/tachycardia	Pooled prevalence estimate, % (95% CI)	7.63 (4.76–12.03)	7.01 (4.36–11.08)	3.35 (1.72–6.44)	6.73 (3.96–11.21)
	I^2^, %	97	92	88	96
	No. of studies, n	20	11	7	13
	COE	Very low	Very low	Very low	Very low
Pain					
Chest pain	Pooled prevalence estimate, % (95% CI)	7.52 (5.22–10.74)	6.02 (3.65–9.77)	4.96 (3.55–6.88)	9.61 (5.94–15.20)
	I^2^, %	97	93	74	95
	No. of studies, n	31	14	6	8
	COE	Very low	Low	Low	Very low
Headache	Pooled prevalence estimate, % (95% CI)	8.12 (6.31–10.39)	8.32 (5.12–13.26)	4.59 (2.42–8.55)	7.30 (4.04–12.85)
	I^2^, %	95	95	91	97
	No. of studies, n	45	13	6	14
	COE	Low	Low	Low	Very low
Arthralgia	Pooled prevalence estimate, % (95% CI)	12.66 (8.79–17.9)	12.66 (6.39–23.52)	11.70 (7.95–16.91)	10.21 (4.81–20.37)
	I^2^, %	98	98	92	97
	No. of studies, n	25	9	6	7
	COE	Very low	Low	Low	Very low
Myalgia	Pooled prevalence estimate, % (95% CI)	13.32 (10.18–17.23)	5.64 (3.13–9.95)	4.31 (2.35–7.79)	12.03 (5.48–24.37)
	I^2^, %	97	93	85	99
	No. of studies, n	34	11	5	11
	COE	Very low	Very low	Low	Very low
Cognitive impairment					
Cognitive impairment (unspecified)	Pooled prevalence estimate, % (95% CI)	6.74 (1.16–30.85)	22.29 (17.98–27.30)	12.66 (5.38–26.99)	10.96 (2.60–36.23)
	I^2^, %	100	13	95	95
	No. of studies, n	8	4	5	2
	COE	Very low	Moderate	Low	Low
Memory disturbance	Pooled prevalence estimate, % (95% CI)	10.42 (6.46–16.41)	12.97 (5.03–29.54)	5.20 (3.51–7.64)	13.07 (3.89–35.82)
	I^2^, %	98	99	0	99
	No. of studies, n	22	10	2	7
	COE	Very low	Very low	Low	Very low
Concentration disturbance	Pooled prevalence estimate, % (95% CI)	15.52 (8.80–25.89)	11.85 (4.37–28.34)	6.39 (3.36–11.81)	29.88 (12.07–56.96)
	I^2^, %	98	99	71	99
	No. of studies, n	15	10	2	6
	COE	Very low	Very low	Low	Low
Brain fog	Pooled prevalence estimate, % (95% CI)	8.47 (2.33–26.42)	9.85 (1.67–41.19)	^c^	2.70 (1.90–3.50)
	I^2^, %	99	99	^c^	NA
	No. of studies, n	5	4	^c^	1
	COE	Very low	Very low	^c^	Low
Clinical psychopathologies					
Anxiety	Pooled prevalence estimate, % (95% CI)	16.68 (12.48–21.95)	23.93 (3.97–70.52)	14.12 (2.99–46.74)	18.33 (13.03–25.16)
	I^2^, %	96	98	98	93
	No. of studies, n	24	4	3	8
	COE	Very low	Low	Very low	Low
Depression	Pooled prevalence estimate, % (95% CI)	17.34 (13.35–22.23)	40.42 (16.35–70.19)	15.71 (7.10–31.27)	18.45 (2.81–63.86)
	I^2^, %	94	93	95	98
	No. of studies, n	20	2	3	3
	COE	Very low	Very low	Very low	Very low
PTSD	Pooled prevalence estimate, % (95% CI)	15.11 (11.18–20.11)	8.59 (5.99–12.17)	14.36 (2.53–51.96)	6.22 (3.2–11.75)
	I^2^, %	93	0	98	84
	No. of studies, n	19	4	2	4
	COE	Low	Low	Very low	Low
Health-related quality of life					
Sleep disturbance (unspecified)	Pooled prevalence estimate, % (95% CI)	16.74 (11.63–23.51)	17.09 (9.74–28.26)	13.78 (7.50–23.97)	29.42 (21.29–39.11)
	I^2^, %	98	96	96	97
	No. of studies, n	23	8	6	10
	COE	Very low	Low	Very low	Low
Insomnia	Pooled prevalence estimate, % (95% CI)	11.79 (7.76–17.52)	10.29 (4.78–20.78)	6.06 (2.13–16.05)	9.93 (4.2–21.71)
	I^2^, %	95	95	92	92
	No. of studies, n	12	5	3	3
	COE	Very low	Very low	Very low	Low
Outcome					
Mobility problems	Pooled prevalence estimate, % (95% CI)	20.58 (3.12–67.62)	31.81 (20.11–46.36)	8.93 (7.31–10.55)	5.98 (2.03–16.34)
	I^2^, %	100	84	NA	93
	No. of studies, n	8	4	1	2
	COE	Very low	Very low	Moderate	Very low
Did not return to work^d^	Pooled prevalence estimate, % (95% CI)	34.86 (23.44–48.33)	36.89 (24.29–51.56)	11.22 (8.96–14.02)	17.27 (8.33–32.41)
	I^2^, %	89	51	0	83
	No. of studies, n	5	2	2	3
	COE	Low	Very low	Moderate	Low
Difficulties performing daily activities	Pooled prevalence estimate, % (95% CI)	20.19 (9.72–37.28)	30.38 (21.26–41.37)	0.37 (0.03–4.77)	8.66 (4.21–17.01)
	I^2^, %	96	92	85	95
	No. of studies, n	10	9	2	6
	COE	Low	Low	Low	Very low
Difficulties with self-care	Pooled prevalence estimate, % (95% CI)	12.72 (3.36–37.94)	23.09 (6.41–56.83)	2.71 (0.74–9.45)	1.27 (0.79–2.03)
	I^2^, %	98	96	90	0
	No. of studies, n	7	4	2	2
	COE	Very low	Very low	Very low	Low
Unable to return to work^d^	Pooled prevalence estimate, % (95% CI)	38.24 (17.80–63.90)	44.29 (32.65–55.92)	33.33 (20.76–45.91)	37.50 (24.82–50.18)
	I^2^, %	93	NA	NA	NA
	No. of studies, n	3	1	1	1
	COE	Very low	Very low	Very low	Very low

**Abbreviations: **CI, confidence interval; COE, certainty of evidence; GRADE, Grading of Recommendations, Assessment, Development, and Evaluations; I2, statistic for assessing heterogeneity; 

NA, not applicable; PCC, post-COVID condition; PTSD, posttraumatic stress disorder. 

^a^ Using total samples. 

^b^ Symptoms and outcomes were selected during consultations with patient representatives, clinical experts and policy makers, and were based on patient concerns, clinical relevance and impact on health care service delivery. 

^c^ No studies were identified for this outcome at this follow-up period.

^d^ “Did not return to work” and “unable to return to work” were reported separately by some studies. Collectively these are described as “limitations in returning to work.” 

For the 12- to 26-week follow-up period, just over half of COVID-19 survivors reported experiencing one or more symptoms (56.50%, 32 studies, very low COE) (see Table 2 and Figure 2a). Fatigue (29.90%, 62 studies, very low COE) was the most prevalent symptom, followed by dyspnea (20.55%, 61 studies, low COE), depression (17.34%, 20 studies, very low COE), unspecified sleep disturbance (16.74%, 23studies, very low COE) and anxiety (16.68%, 24 studies, very low COE). Limitations in returning to work was the most prevalent functional outcome during this period (see Table 2 and Figure 2b). 

**Figure 2a:**
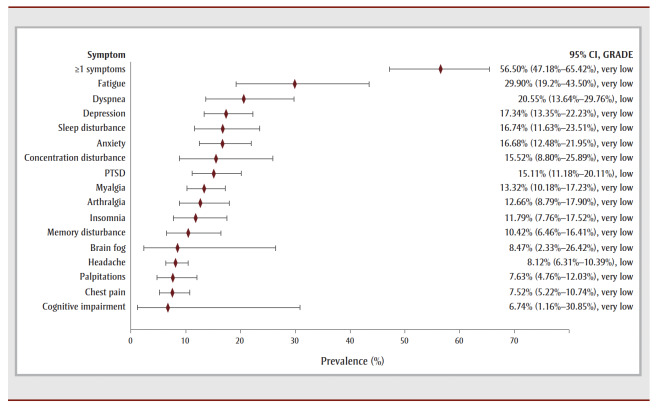
Pooled prevalence estimates of PCC symptoms at 12–26 weeks after confirmed COVID-19

**Figure 2b:**
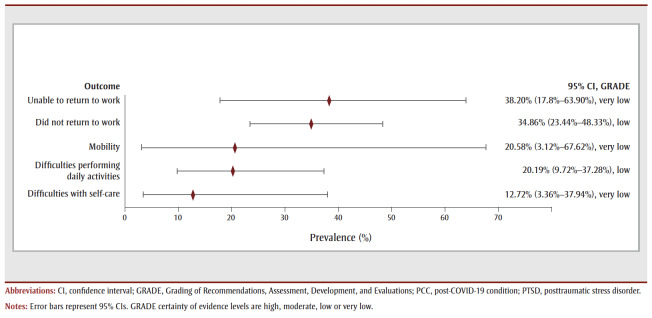
Pooled prevalence estimates of PCC outcomes at 12–26 weeks after confirmed COVID-19

For the 27- to 39-week follow-up period, half of the COVID-19 survivors experienced one or more symptoms (50.89%, 9 studies, very low COE) (see Table 2 and Figure 3a). Depression was the most prevalent symptom (40.42%, 2 studies, very low COE) followed by fatigue (28.38%, 25studies, very low COE), anxiety (23.93%, 4 studies, low COE), unspecified cognitive impairment (22.29%, 4 studies, moderate COE), unspecified sleep disturbance (17.09%, 8 studies, low COE) and dyspnea (14.81%, 22 studies, low COE). Limitations in returning to work was the most prevalent functional outcome during this period (see Table 2 and Figure 3b).

**Figure 3a:**
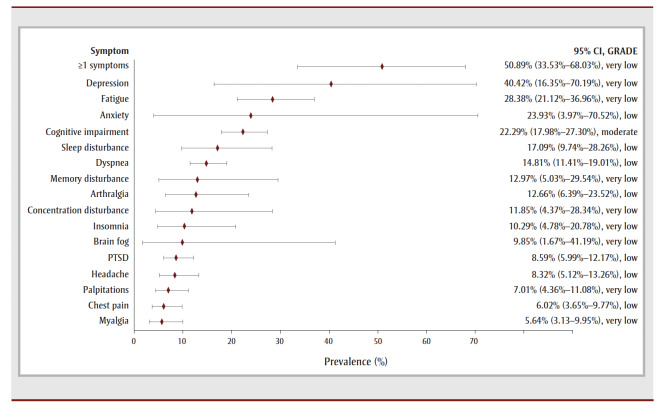
Pooled prevalence estimates of PCC symptoms at 27–39 weeks after confirmed COVID-19

**Figure 3b:**
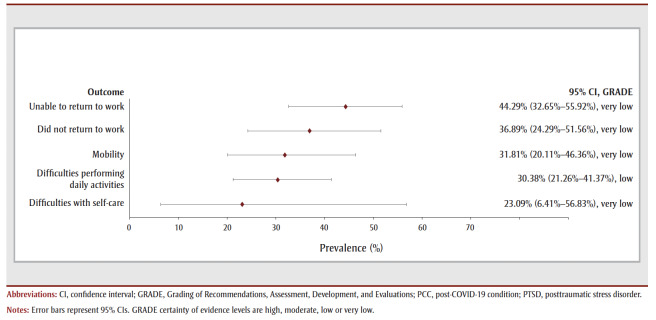
Pooled prevalence estimates of PCC outcomes at 27–39 weeks after confirmed COVID-19

For the 40- to 52-week follow-up period, nearly one-third of COVID-19 survivors reported experiencing one or more symptoms (32.64%, 4 studies, low COE) (see Table 2 and Figure 4a. The most prevalent symptom was fatigue (30.70%, 12studies, low COE) followed by dyspnea (16.06%, 15 studies, low COE), depression (15.71%, 3 studies, very low COE), PTSD (14.36%, 2 studies, very low COE), anxiety (14.12%, 3 studies, very low COE) and unspecified sleep disturbance (13.78%, 6 studies, very low COE). The most prevalent functional outcomes were limitations in returning to work and mobility problems (see Table 2 and Figure 4b).

**Figure 4a:**
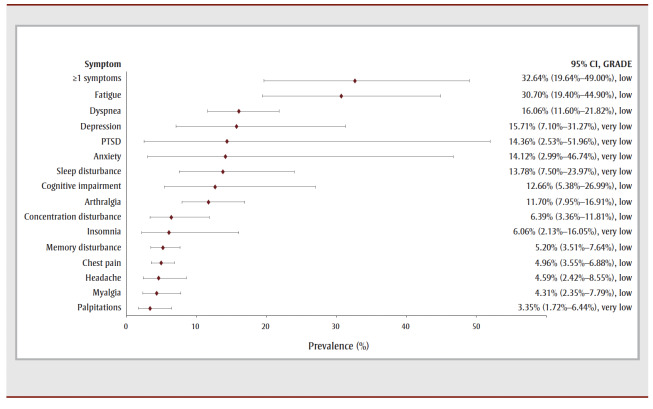
Pooled prevalence estimates of PCC symptoms at 40–52 weeks after confirmed COVID-19

**Figure 4b:**
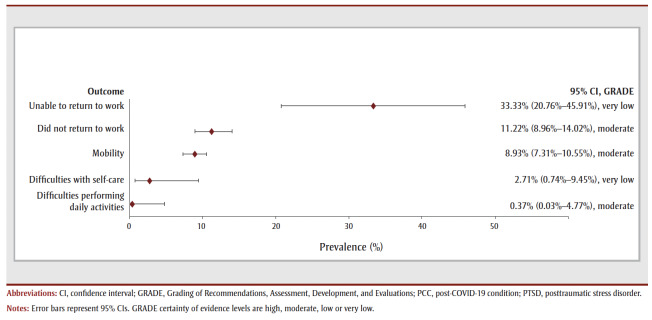
Pooled prevalence estimates of PCC outcomes at 40–52 weeks after confirmed COVID-19

In the follow-up period of more than 1 year, most COVID-19 survivors experienced one or more symptoms (77.64%, 6 studies, very low COE) (see Table 2 and Figure 5a). The most prevalent symptom was concentration disturbance (29.88%, 6 studies, low COE) followed by sleep disturbance (29.42%, 10 studies, low COE), fatigue (26.90%, 19 studies, very low COE), depression (18.45%, 3 studies, very low COE), anxiety (18.33%, 8 studies, low COE) and dyspnea (15.62%, 18 studies, very low COE). The most prevalent functional outcomes were inability to return to work (37.50%, 1 study, very low COE), not returning to work (17.27%, 3 studies, low COE) and difficulties performing daily activities (8.7%, 6 studies, very low COE) (see Table 2 and Figure 5b).

**Figure 5a:**
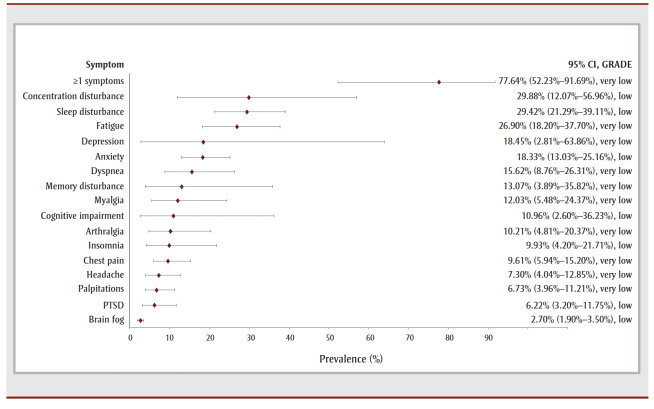
Pooled prevalence estimates of PCC symptoms at more than 1 year after confirmed COVID-19

**Figure 5b:**
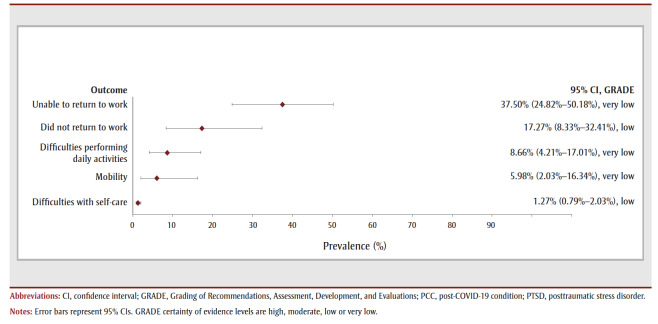
Pooled prevalence estimates of PCC outcomes at more than 1 year after confirmed COVID-19

***Studies involving children and adolescents***

Two included studies explored PCC prevalence in children and adolescents (≤18years) only.^185^,^214^ Fatigue was the most common symptom, with prevalence from 10.7% to 14.6% at more than 4 to 5 months of follow-up. 185,214 Osmanov et al.^214^ also reported increased prevalence of sleep disturbance (6.9%) and sensory problems (5.6%) among previously hospitalized children at more than 5 months of follow-up. Pazukhina et al.^215^ examined all age groups and reported a PCC prevalence of 20% in children (median age: 9.5 years) at the 6-month follow-up, with fatigue the most common symptom (9%). 

***Subgroup analyses***

Results of the subgroup analyses suggest that certain populations may have experienced a greater PCC burden than others (see tables and forest plots in Supplementary Material II). Higher point prevalence was reported among females than males for most symptoms and functional outcomes, with the exception of anxiety, depression, arthralgia, insomnia and mobility problems for some follow-up periods, although often these were not statistically significant (i.e. the confidence intervals overlapped for the point estimates). Higher pooled prevalence was also often observed with increasing severity of the disease or with hospitalization or ICU admission during the initial infection. However, in some cases those who were not hospitalized had higher point prevalence of mobility problems, difficulties with self-care, pain and sleep disturbance. In addition, many of these comparisons were not statistically significant.

## Discussion

This systematic review includes 194 prospective studies that explored the prevalence of selected PCC symptoms and outcomes. Together, these studies examined a total of 483 531 individuals with confirmed COVID-19 over follow-up periods of up to 2 years. This review is the first to stratify reported prevalence data in four distinct follow-up periods after the initial infection to examine the trajectory of PCC prevalence over time.

At least half of the COVID-19 survivors reported one or more PCC symptoms across nearly all follow-up periods. The pooled prevalence estimate for one or more symptoms was highest for the more-than-1-year period. However, whether certain sample characteristics (e.g. higher proportion of hospitalized patients) influenced this increase could not be fully explored because of the limited number of studies contributing to this outcome.

Our analysis showed that fatigue (26.90%–30.70%), dyspnea (14.81%–20.55%), clinical psychopathologic symptoms (6.22%– 40.42%), sleep disturbance (13.78%–29.42%), memory disturbance (5.20%–13.07%), concentration disturbance (6.39%–29.88%) and pain (4.31%–13.32%) were the most prevalent symptoms. Although subgroup analyses could not be performed for all follow-up periods because of the insufficient number of studies, we often observed higher point prevalence (not always statistically significantly higher) in the following subgroups: females; individuals who were hospitalized or admitted to the ICU during acute illness; and individuals who experienced severe COVID-19.

The lower PCC prevalence among children and adolescents compared to adults was evident across the few identified studies that examined this age group,^185^,^214^,^215^,^228^ which aligns with other systematic reviews and reports.^5^,^6^,^229^ These publications reported that common symptoms among children include fatigue, anosmia, headache, anxiety, anorexia, earache/tinnitus and sore eyes.^5^,^6^,^229^ A 2021 population survey conducted in the United Kingdom found self-reported prevalence of PCC to range from 0.16% among those aged 2 to 11 years to 0.65% among those aged 12 to 16 years and 1.22% among those aged 17 to 24 years.^6^,^230^

Most of the studies in this review examined only hospitalized populations, which precludes drawing useful comparisons with healthy control groups. As a result, our prevalence estimates may be overestimated, as we could not adjust for baseline prevalence rates of non-PCC-related symptoms. In addition, more than two-thirds of the study populations were in European countries, and there was less information on PCC prevalence in other parts of the world. 

Many studies did not report major risk factors such as age, sex, race or ethnicity, socioeconomic status or pre-existing health conditions, and thus were omitted from the subgroup analyses. This lack of reporting hindered our ability to compare PCC prevalence between males and females or children and adults, for example. We also observed considerable variations in the ways symptoms and outcomes were defined or assessed across the studies. 

The heterogeneity of each outcome across the included studies varied widely, with 40% of analyses demonstrating levels of 75% or greater. When investigating potential sources of bias, we noted concerns to do with sampling of study participants, adequacy of participants’ response rates and approaches to managing low response rates. We also noted biases regarding the validity of methods used to diagnose COVID-19 and to assess the symptoms and outcomes across different studies.

In-depth GRADE assessment showed that 99% of the outcomes analyzed had serious or very serious ROB. Limiting the assessments to studies with low to moderate ROB would have substantially enhanced the overall GRADE levels. In nearly one-third of analyses, heterogeneity could be explained in part by one or more subgroup analyses. Indirectness was deemed serious in 90% of analyses because of the predominant focus on hospitalized populations, which may have contributed to higher prevalence estimates.

Results of the 2023 Canadian COVID-19 Antibody and Health Survey (CCAHS) revealed that nearly 20% of COVID-19 survivors (6.8% of adults in Canada) experienced PCC symptoms.^16^ Of this group, nearly 80% continued to experience these symptoms for 6 months or longer, and more than 40% for a year or longer.^16^ Earlier results reported that prevalence was higher among females, those initially hospitalized for severe COVID-19 and individuals with pre-existing chronic conditions.^18^ Common symptoms reported from Cycle 1 of the survey included fatigue (72.1%), dyspnea (38.5%) and brain fog (32.9%).^231^

A similar recent US survey found that approximately 14.3% of adults reported experiencing PCC symptoms, with higher prevalence among females and younger individuals.^15^ A survey of private households in the United Kingdom found that 2.6% of the total population self-reported PCC symptoms for at least 12 weeks, with 69% continuing to experience the symptoms for a year or longer.^11^ An Australian population survey reported that 9.7% of individuals with confirmed or suspected COVID-19 (4.7% of all adults) experienced PCC symptoms.^17^

Our examination of these recent systematic reviews and reports suggests that PCC prevalence ranged between 2.5% and 63.9%.^10^,^12^,^16^,^232^-^244^ This wide range can be attributed to several factors including, but not limited to, the pooling of confirmed and suspected cases, the combination of various study types (prospective and retrospective studies including cross-sectional studies, case reports and case series) and the sampling approach (hospitalized, community-based patients or both). 

Other factors include inconsistencies in the definition of PCC, the pooling of studies with different follow-up durations and the diverse methods used to assess symptoms and outcomes with varying degrees of validity. In addition, the variability in demographic characteristics of the participants included in population surveys may have also contributed to this wide variation in prevalence estimates.

The most frequently reported symptoms identified through our extended search were dyspnea (5.4%–80.6%)^12^,^233^-^235^,^237^,^242^,^244^-^248^ and fatigue (9.3%–54.2%)^12^,^233^-^237^,^242^,^244^-^250^. Others, in order of frequency, were disturbance in health-related quality of life (1.0%–52.0%),^12^,^235^,^244^,^247^,^248^ sleep disturbance (3.5%–47.4%)^12^,^233^-^235^,^237^,^239^,^242^,^244^,^245^,^249^,^251^,^252^ and pain (1.0%–34.5%).^12^,^233^-^236^,^245^,^247^,^248^,^250^ The collective prevalence of anxiety, depression and PTSD ranged from 2.0% to 32.0%^12^,^233^-^237^,^239^,^242^,^244^,^245^,^248^,^250^,^252^; of functional impairment from 4.0% to 36.0%^12^,^235^,^244^,^247^; of cognitive impairment from 13.5% to 30%^12^,^248^,^249^; of brain fog from 25.5% to 36.0%^242^,^248^,^250^; of concentration disturbance from 8.0% to 29%^12^,^233^-^235^,^239^,^244^,^245^,^248^; and of palpitations from 1.0% to 23.0%^12^,^233^-^236^,^239^,^242^,^244^,^245^,^248^. These findings, derived from reports and systematic reviews included in the extended search, align with our primary findings, particularly regarding the higher prevalence of PCC symptoms among females and among individuals with a history of hospitalization or ICU admission during their initial infection. Data from a 2024 National Academies report^228^ were also in line with our findings, as were the findings in the systematic reviews^12^,^232^-^239^,^242^,^245^-^252^ and authoritative reports^10^,^231^,^240^-^244^ identified in our extended search.

***Strengths and limitations***

This is the first systematic review of evidence that focuses exclusively on prospective studies. We opted to focus on these higher-quality studies to strengthen the reliability of our findings, even if doing so meant foregoing valuable insights from retrospective evidence.

The review summarizes the published evidence on PCC prevalence from various population groups and health care systems over follow-up periods of up to 2years. The extended search differentiates it from previous reviews that focused on the earlier stages of the condition. 

Restricting the study search to English or French language publications (due to time and resources constraints) is a potential limitation. However, we anticipate minimal impact on the overall yield of the search based on prior evidence that language restrictions in systematic reviews often have minimal impact on the overall yield of high-quality evidence, particularly in fields where the majority of relevant and high-quality studies are published in English or French and indexed in the databases we used.^253^

We used a version of the GRADE approach, modified in cooperation with GRADE experts, to better fit the current review when evaluating the COE and assessing the level of confidence in the reported findings. Although this modified version has not yet been validated, it has been adopted for use in other studies (e.g. Righy et al.^33^). GRADE was originally designed for studies of therapeutic interventions, and it continues to present challenges when applied to studies of nontherapeutic exposures or prognostic factors^254^ and for prevalence studies. Compared to randomized controlled trials, observational studies are often more heterogenous because of variations in study design, population and sampling as well as nonstandardized outcome assessments. This inherent variability frequently leads to a downgrading of level of evidence certainty, as in our review, where high heterogeneity occurred in nearly 61% of the analyses.

In this review, we did not examine PCC prevalence in undiagnosed individuals or those with suspected but unconfirmed COVID-19. A critical consideration in interpreting PCC prevalence estimates is the type of population included in these studies. Population-based studies that focus solely on PCR-confirmed infections typically report higher prevalence rates (20%–25%), reflecting symptomatic and more severe cases.^232^,^255^ In contrast, studies that incorporate all cases, even asymptomatic cases identified through serological surveys, generally present lower prevalence rates (5%–10%).^232^,^255^ This discrepancy highlights the selective nature of PCR testing during high-demand periods, which predominantly captured symptomatic infections. The current review primarily included prospective studies with confirmed infections, and we acknowledge that this approach might not fully capture PCC prevalence, particularly among asymptomatic or undiagnosed cases.

The differences between our findings and those of population surveys identified in our extended search, such as the CCAHS, likely arise from variances in respondent characteristics, inclusion of both confirmed and suspected cases, and the subjective nature of outcome assessment. These population surveys, although widely used, are prone to sampling bias, self-reporting bias, nonresponse bias and other limitations that can influence prevalence estimates. Furthermore, the temporal relationship between COVID-19 and the reported PCC symptoms may not always be clear, adding to the variability in prevalence data. By comparing our results with these surveys, we aim to highlight how methodological differences and biases in population surveys can account for the observed variations in prevalence estimates.

Our review primarily focused on assessing the global prevalence of PCC to inform clinicians and policy makers. Accordingly, we did not explore any influence of COVID-19 vaccines or the different SARS-CoV-2 variants on PCC prevalence. Expanding the analysis to include such variables would have significantly increased the scope and complexity, exceeding our resources. Also, the lack of consistent reporting on vaccination and voice-of-the-customer (VOC) data across studies would have introduced potential inaccuracies and inconsistencies if inferred from external sources.

Our pooled prevalence estimates were derived from diverse patient cohorts across various follow-up periods, rather than a single continuously monitored cohort. Therefore, it is essential to carefully evaluate the presented synthesis of evidence while taking into account its strengths and limitations. 

***Suggestions for future research***

The current review highlights the importance of examining PCC prevalence based on major risk factors; and on standardizing outcome assessment methods and case management protocols. Moreover, we encourage prioritizing investigations into equity-deserving populations because certain population groups experienced and continue to experience more pronounced PCC effects.

## Conclusion

This review contributes to our collective understanding of the global burden of PCC. Many COVID-19 survivors continue to experience symptoms and functional impairments more than a year after their initial infection. The most commonly reported symptoms include fatigue and dyspnea, which aligns with other published reviews and reports. As emphasized in an earlier version of this systematic review,^256^ these results are intended to amplify patient voices, aid researchers and clinicians, and guide policy makers and decision makers in the development of mitigation strategies and support services for COVID-19 survivors living with PCC and their caregivers.

## Acknowledgements

We would like to acknowledge the invaluable contributions of our colleagues and team members: Lynda Gamble (library search), Yi Xuan Wang (data abstraction) and Maria Benkhalti, Meghan Grainger and Veronica Belcourt (risk of bias assessment). We also appreciate their help with reviewing this manuscript. We also would like to thank our external experts, Dr. Adrienne Stevens (McMaster University, Hamilton, ON, Canada), Dr. Maicon Falavigna (Hospital de Clnicas de Porto Alegre, Porto Alegre, Rio Grande do Sul, Brazil) and Dr. Zachary Munn (Health Evidence Synthesis, Recommendations and Impact [HESRI], School of Public Health, University of Adelaide, South Australia, Australia) for their help in adapting the JBI checklist and the GRADE approach for use in the present review.

## Funding

The Public Health Agency of Canada.

## Conflicts of interest

None to declare.

## Authors’ contributions and statement

MKT: Data curation, formal analysis, investigation, project administration, supervision, visualization, writing – original draft, writing – review and editing.

TS: Data curation, investigation, validation, visualization, writing – original draft.

AB: Data curation, investigation, writing – original draft.

KM: Conceptualization, data curation, investigation, validation, writing – review and editing.

FRD: Conceptualization, data curation, investigation, validation, writing – review and editing.

AMC: Conceptualization, writing – review and editing.

CLC: Conceptualization, writing – review and editing.

LB: Conceptualization, data curation, investigation, validation, writing – review and editing.

AMZ: Conceptualization, data curation, investigation, validation, writing – review and editing.

MAM: Conceptualization, formal analysis, software, visualization, writing – review and editing.

CL: Conceptualization, data curation, investigation, validation, writing – review and editing.

RC: Data curation, investigation, validation, writing – review and editing.

AH: Data curation, investigation, validation, writing – review and editing.

PR: Investigation, validation, writing – review and editing.

EC: Data curation, investigation, writing – review and editing.

TC: Conceptualization, investigation, validation, writing – review and editing.

LAW: Conceptualization, writing – review and editing.

JEP: Data curation, investigation, writing – review and editing.

RA: Conceptualization, writing – review and editing.

AJG: Conceptualization, writing – review and editing.

All the authors conducted a thorough review of the manuscript, provided feedback and approved the final manuscript. All authors had access to the study data.

The content and views expressed in this article are those of the authors and do not necessarily reflect those of the Government of Canada.
