# Global prevalence of post-COVID-19 condition: a systematic review and meta-analysis of prospective evidence

**DOI:** 10.24095/hpcdp.45.6.06

**Published:** 2025-06

**Authors:** Mohamed Kadry Taher, Talia Salzman, Allyson Banal, Kate Morissette, Francesca R. Domingo, Angela M. Cheung, Curtis L. Cooper, Laura Boland, Alexandra M. Zuckermann, Muhammad A. Mullah, Claudie Laprise, Roberto Colonna, Ayan Hashi, Prinon Rahman, Erin Collins, Tricia Corrin, Lisa A. Waddell, Jason E. Pagaduan, Rukshanda Ahmad, Alejandra P. Jaramillo

**Affiliations:** 1 Evidence Synthesis and Knowledge Translation Unit, Centre for Surveillance and Applied Research, Health Promotion and Chronic Disease Prevention Branch, Public Health Agency of Canada,; 2 School of Epidemiology and Public Health, University of Ottawa, Ottawa, Ontario, Canada; 3 Department of Medicine and Joint Department of Medical Imaging, University Health Network and Sinai Health System, University of Toronto, Toronto, Ontario, Canada; 4 Toronto General Hospital Research Institute and Schroeder Arthritis Institute, Toronto, Ontario, Canada; 5 Department of Medicine, University of Ottawa; Ottawa Hospital Research Institute, Ottawa, Ontario, Canada; 6 Infectious Disease and Vaccination Programs Branch, Centre for Communicable Diseases and Infection Control, Public Health Agency of Canada, Ottawa, Ontario, Canada; 7 Department of Social and Preventive Medicine, School of Public Health, Universit de Montral, Montral, Quebec, Canada; 8 Population Health Modelling Unit, Centre for Surveillance and Applied Research, Health Promotion and Chronic Disease Prevention Branch, Public Health Agency of Canada, Ottawa, Ontario, Canada; 9 Public Health Risk Sciences Division, National Microbiology Laboratory, Public Health Agency of Canada, Guelph, Ontario, Canada; 10 Risk Assessment Division, Centre for Surveillance, Integrated Insights and Risk Assessment, Data, Surveillance and Foresight Branch, Public Health Agency of Canada, Ottawa, Ontario, Canada

This corrigendum is being published to correct a number of errors and imprecisions, on pages 113, 120–125 and 138, of the following article: 

Taher MK, Salzman T, Banal A, Morissette K, Domingo FR, Cheung AM, Cooper CL, Boland L, Zuckermann AM, Mullah MA, Laprise C, Colonna R, Hashi A, Rahman P, Collins E, Corrin T, Waddell LA, Pagaduan JE, Ahmad R, Jaramillo Garcia AP. Global prevalence of post-COVID-19 condition: a systematic review and meta-analysis of prospective evidence. Health Promot Chronic Dis Prev Can. 2025;45(3):112-38. https://doi.org/10.24095/hpcdp.45.3.02 

The authors would like to clarify a few points specifically related to the referencing of results from the 2023 Canadian COVID-19 Antibody and Health Survey (CCAHS).^1^ These clarifications reflect refinements in how the source data are interpreted and attributed, and do not affect the core findings or conclusions of the review. Bold has been used to identify the changes and updated text. 

## 1. p. 113, middle column, paragraph 2:

***Before correction***

Results from recent population surveys conducted to assess the overall prevalence of PCC symptoms among adults vary from 14.3% in the USA^15^ to 6.8% in Canada^16^ and 4.7% in Australia.^17^

***After correction***

Results from recent population surveys conducted to assess the **prevalence** of PCC symptoms among adults vary from 14.3% in the USA^15^ to **11.7%** in Canada^16^ and 4.7% in Australia.^17^

## 2. p. 120, last column, paragraph 3 and p. 125, first column, paragraph 1:

The changes made in the following paragraph include the addition of a new reference, numbered 257 for convenience. The statement regarding higher PCC prevalence among females, individuals hospitalized during acute infection and those with pre-existing chronic conditions was previously attributed only to a Statistics Canada table which did not include all of those breakdowns. The new reference (see item 3, below) contains the comprehensive data supporting this statement. 

***Before correction***

Results of the 2023 Canadian COVID-19 Antibody and Health Survey (CCAHS) revealed that nearly 20% of COVID-19 survivors (6.8% of adults in Canada) experienced PCC symptoms.^16^ Of this group, nearly 80% continued to experience these symptoms for 6 months or longer, and more than 40% for a year or longer.^16^ Earlier results reported that prevalence was higher among females, those initially hospitalized for severe COVID-19 and individuals with preexisting chronic conditions.^18^ Common symptoms reported from Cycle 1 of the survey included fatigue (72.1%), dyspnea (38.5%) and brain fog (32.9%).^231^

***After correction***

Results of the 2023 Canadian COVID-19 Antibody and Health Survey (CCAHS) revealed that nearly 20% of COVID-19 survivors experienced PCC symptoms.^16^ **This corresponds to 11.7% of the total adult population, or approximately 3.5 million Canadians. Among those who experienced PCC symptoms during the time of the survey (6.8%)**, nearly 80% continued to experience these symptoms for 6 months or longer, and more than 40% for a year or longer.^16^ Earlier results reported that prevalence **of PCC among COVID-19 survivors** was higher among females, those initially hospitalized for severe COVID-19 and individuals with preexisting chronic conditions.^18^,^257^ Common symptoms reported from **Cycle 2** of the survey included fatigue (72.1%), dyspnea (38.5%) and brain fog (32.9%).^231^

## 3. p. 138, new reference:

**257. Government of Canada. COVID-19: Longer-term symptoms among Canadian adults – Second report [Internet]. Ottawa (ON): Health Infobase Canada; [updated 2024 Aug 21; cited 2025 May 13]. Available from: https://health-infobase.canada.ca/covid-19/post-covid-condition/spring-2023-report.html#a6**

## References

[B01] Kuang S, Earl S, Clarke J, Zakaria D, Demers A, Aziz S (2023). Experiences of Canadians with longterm symptoms following COVID-19. Statistics Canada.

